# Cerebrospinal fluid GFAP is a predictive biomarker for conversion to dementia and Alzheimer’s disease-associated biomarkers alterations among de novo Parkinson’s disease patients: a prospective cohort study

**DOI:** 10.1186/s12974-023-02843-5

**Published:** 2023-07-20

**Authors:** Tingting Liu, Hongzhou Zuo, Di Ma, Dan Song, Yuying Zhao, Oumei Cheng

**Affiliations:** Department of Neurology, The First Affiliated Hospital of Chongqing Medical University, Chongqing, 400016 China

**Keywords:** Parkinson’s disease, Cerebrospinal fluid, Glial fibrillary acidic protein, Parkinson’s Progression Markers Initiative, Cognition decline

## Abstract

**Background:**

Dementia is a prevalent non-motor manifestation among individuals with advanced Parkinson’s disease (PD). Glial fibrillary acidic protein (GFAP) is an inflammatory marker derived from astrocytes. Research has demonstrated the potential of plasma GFAP to forecast the progression to dementia in PD patients with mild cognitive impairment (PD–MCI). However, the predictive role of cerebrospinal fluid (CSF) GFAP on future cognitive transformation and alterations in Alzheimer’s disease (AD)-associated CSF biomarkers in newly diagnosed PD patients has not been investigated.

**Methods:**

210 de novo PD patients from the Parkinson’s Progression Markers Initiative were recruited. Cognitive progression in PD participants was evaluated using Cox regression. Cross-sectional and longitudinal associations between baseline CSF GFAP and cognitive function and AD-related CSF biomarkers were evaluated using multiple linear regression and generalized linear mixed model.

**Results:**

At baseline, the mean age of PD participants was 60.85 ± 9.78 years, including 142 patients with normal cognition (PD–NC) and 68 PD–MCI patients. The average follow-up time was 6.42 ± 1.69 years. A positive correlation was observed between baseline CSF GFAP and age (β = 0.918, *p* < 0.001). There was no statistically significant difference in baseline CSF GFAP levels between PD–NC and PD–MCI groups. Higher baseline CSF GFAP predicted greater global cognitive decline over time in early PD patients (Montreal Cognitive Assessment, β = − 0.013, *p* = 0.014). Furthermore, Cox regression showed that high baseline CSF GFAP levels were associated with a high risk of developing dementia over an 8-year period in the PD–NC group (adjusted HR = 3.070, 95% CI 1.119–8.418, *p* = 0.029). In addition, the baseline CSF GFAP was positively correlated with the longitudinal changes of not only CSF α-synuclein (β = 0.313, *p* < 0.001), but also CSF biomarkers associated with AD, namely, amyloid-β 42 (β = 0.147, *p* = 0.034), total tau (β = 0.337, *p* < 0.001) and phosphorylated tau (β = 0.408, *p* < 0.001).

**Conclusions:**

CSF GFAP may be a valuable prognostic tool that can predict the severity and progression of cognitive deterioration, accompanied with longitudinal changes in AD-associated pathological markers in early PD.

**Supplementary Information:**

The online version contains supplementary material available at 10.1186/s12974-023-02843-5.

## Background

Parkinson’s disease (PD) is a chronic neurodegenerative disorder with diverse etiologies and clinical presentations [1]. Among the disabling non-motor symptoms experienced by those with PD, cognitive dysfunction is one of the most prevalent [2]. The prevalence of PD with mild cognitive impairment (PD–MCI) is approximately 40% [3]. The prevalence of PD with dementia (PD–D) ranges from 24 to 31%, reaching 83% within two decades of follow-up, which has a tremendous impact on patients' families and socioeconomics [4, 5]. Furthermore, a scarcity of efficacious therapeutic interventions exists for the cognitive impairments correlated with PD [2]. Therefore, the identification of a reliable biomarker capable of predicting cognitive alterations linked to PD would be an important tool for guiding clinical management and trials [6].

The pathophysiological mechanisms that contribute to cognitive disorder in PD are intricate [7]. Kouli et al. have noted that neuroinflammation is emerging as a momentous pathological factor of PD–D [8]. Astrocytes, the most abundant type of glial cells found in the central nervous system (CNS), are involved in CNS inflammation and are thought to have a vital role in the development of PD and cognitive impairment [9–11].

Glial fibrillary acidic protein (GFAP), a type-III intermediate filament that is predominantly expressed by astrocytes, serves as an indicator of reactive astrogliosis. Its expression is substantially elevated in neurodegenerative illness, including Alzheimer’s disease (AD) and PD [12–14]. Pereira et al. reported that cerebrospinal fluid (CSF) and plasma GFAP were predictors of longitudinal cognitive deterioration in the whole cohort, comprising AD patients, even after correction for longitudinal amyloid-β positron emission tomography (Aβ-PET) changes [15]. Bartl et al. found a possible correlation between CSF GFAP concentration at baseline and Montreal Cognitive Assessment (MoCA) score at 6-year follow-up in newly diagnosed unmedicated PD patients [16]. Tang and colleagues demonstrated that baseline plasma GFAP exhibited a significant negative correlation with Mini-Mental State Examination scores and further served as a predictive factor for the progression from MCI to dementia among patients with PD [17]. In sum, GFAP presents as a potential indicator of cognitive impairment in PD. However, existing studies exhibit certain limitations, including comparatively diminutive sample size, unadjusted confounding factors or insufficient adjustments, and unclarified mechanisms. Consequently, a more comprehensive assessment is required to determine the precise association between GFAP and cognitive function in PD, as well as its predictive capacity.

Several post-mortem investigations have evidenced that the aggregation of Lewy bodies composed of α-synuclein (α-syn) within the limbic system and neocortex represents a principal contributor to cognitive dysfunction in PD [18, 19]. Simultaneously, cognitive decline in PD has also been substantially linked to pathological markers characteristic of AD, specifically Aβ and tau [20–22]. Recent investigations have demonstrated that in AD, GFAP not only correlates with horizontal and vertical alterations in Aβ and tau, but also is partially involved in the effect of Aβ on tau [15, 23, 24]. However, the impact of GFAP on the longitudinal changes in these markers among PD patients remains uncertain.

CSF is the preferred biomarker source for research compared to blood owing to its proximity to the brain structure and its ability to accurately reflect the pathological processes taking place in the brain [25]. Therefore, we hypothesize that CSF GFAP may also predict cognitive deterioration and AD-associated pathological alternations in PD patients. In this study, we aimed to undertake cross-sectional and longitudinal assessments in a cohort of newly diagnosed untreated PD patients, with a follow-up period of 8 years. The primary objective is to determine the correlation between CSF GFAP and cognitive decline across various domains, as well as AD-associated CSF biomarkers. The secondary objective is to speculate the potential pathogenic mechanisms underlying cognitive dysfunction in PD via CSF GFAP.

## Methods

### Study participants

Research data were downloaded from the Parkinson's Progression Markers Initiative (PPMI) repository [26]. The PPMI is a continuing, prospective, longitudinal, observational, and multinational multi-center research program designed to detect biomarkers for PD [27]. The PPMI investigation was sanctioned by the ethics review board of all collaborating establishments; furthermore, all subjects provided their signature on a written informed consent document prior to enrollment.

PD subjects were recruited based on the inclusion criteria: individuals must be 30 years or older at diagnosis; have bradykinesia combined with resting tremor, rigidity, or only asymmetric resting tremor or bradykinesia; be untreated for PD, particularly without medications that might interfere with dopamine transporter imaging or CSF collection; and be free of dementia at baseline (MoCA ≥ 22). To prevent misdiagnosis, the researchers reviewed the diagnosis longitudinally. Patients diagnosed with conditions other than primary PD were excluded from the follow-up. In addition, our investigation mandated that all subjects possess baseline CSF GFAP data. All enrolled participants were evaluated periodically to acquire clinical information and partake in CSF biomarker investigations. Additional file 1: Table S1 shows the clinical and biomarker information of recruited PD patients during follow-up. Longitudinal follow-up included seven yearly assessments of CSF biomarker and eight yearly clinical evaluations subsequent to the initial lumbar puncture.

### Measurement of CSF GFAP

The collection and processing of CSF biomarker were conducted in accordance with the PPMI biologics manual. CSF GFAP concentrations were quantified by Roche NeuroToolKit (NTK) on a cobas e 411 analyzer at Covance Greenfield Laboratories (Translational Biomarker Solutions, Indiana, USA) [16].

### Measurement of CSF biomarkers

CSF Aβ42, total tau (T-tau) and phosphorylated tau (P-tau) concentrations were quantified through an Elecsys^®^ electrochemiluminescence immunoassay (ECLIA) using a completely automated cobas e 601 analyzer (Roche Diagnostics, Basel, Switzerland) [28]. The levels of CSF α-syn were determined utilizing a suitable commercially available sandwich ELISA kit (Covance, Dedham, MA) [29].

### Genotyping

Genomic DNA was extracted from the whole blood of PD subjects for processing and analysis. Apolipoprotein E (APOE) genotypes were detected by allele-specific oligonucleotide probes labeled with a fluorogenic reporter (TaqMan method) [29]. Besides, individuals were also divided into two groups according to the ε4 allele, namely, APOE ε4 carriers (APOE ε4 +) and non-APOE ε4 carriers (APOE ε4 −).

### Clinical assessment measures

The cognitive assessment scales included in this study were as follows: global cognitive functioning (MoCA), episodic memory (Hopkins Verbal Learning Test, HVLT), visuospatial abilities (Benton Judgment of Line Orientation, JoLO), executive functioning/working memory (Letter Number Sequencing, LNS), language (Semantic Fluency Test), and processing speed/attention (Symbol Digit Modalities Test, SDMT). Cognitive status of participants was classified based on the criteria used in previous studies [30, 31]: normal cognition (NC, MoCA > 26), MCI (22 ≤ MoCA ≤ 26), dementia (MoCA < 22).

### Statistical analysis

CSF GFAP levels did not distribute normally (Kolmogorov–Smirnov test, *p* < 0.05) and hence were log10-transformed to approach the normal distribution (Additional file 1: Fig. S1). Log10-transformed values were used in all analyses in this study. Continuous demographic, clinical assessment and biomarker data were compared using ANOVA. Chi-square tests were used for qualitative variables. Differences in CSF GFAP concentrations between diagnostic subgroups were analyzed by Student’s *t* tests.

Multiple linear regression models were employed to evaluate the baseline correlation of GFAP with CSF biomarkers and cognitive characteristics. In parallel, generalized linear mixed models (GLMM) were utilized to assess the effect of baseline GFAP level or the interaction of baseline GFAP level with follow-up time on other measured longitudinal data. We analyzed models with tertile and continuous variables. Mediating effect analysis was used to determine whether CSF GFAP mediates between CSF Aβ42 and T-tau or P-tau. Due to the shared neurological spectrum between PD and AD [32], the AD-associated CSF profiles in PD were characterized by applying cutoff values for Aβ42 and P-tau proteins. To reduce variations in pre-analytical factors that affect CSF Aβ42 levels among both PPMI and AD cohorts [33], we undertook the conversion of Elecsys values into AlzBio3 equivalent values [x = (CSF Aβ42 + 251.55)/3.74] through the utilization of a conversion formula and considered a dependable cut-point of AlzBio3 corresponding values (< 250 pg/ml) that could effectively distinguish a positive or negative amyloid status [28, 34]. Moreover, positive status of tau was defined as P-tau > 21.8 pg/ml according to previous studies [35].

Next, the cumulative incidence of cognitive progression among various groups during follow-up was compared using Kaplan–Meier survival curves. In addition, the relationship between baseline CSF GFAP level and cognitive stage transition during follow-up was analyzed using multivariate Cox regression models.

The statistical analyses in this study were conducted via SPSS 25.0. All regression analyses and GLMM were subject to adjustments for confounding variables, including age, gender, education level, APOE ɛ4 carrier status, and disease duration. In addition, to exclude the effect of CSF biomarkers on PD cognition, we also included α-syn, Aβ42 and P-tau in the analysis.

## Results

### Study participants

The demographic and clinical characteristics of the study cohort, comprising 210 individuals with de novo PD, are presented in Table 1. The average age at baseline for the whole cohort was 60.85 ± 9.78 years, with a male proportion of 64.8%. The mean duration of disease was 7.41 ± 6.97 months, while the average education level was 16.12 ± 2.62 years. The average follow-up time was 6.42 ± 1.69 years. Among the participants, 53 (27.2%) were carriers of the APOE ε4 allele.

**Table 1:** Baseline clinical characteristics and CSF biomarkers of PD participants in this study

Characteristics	Patients with PD (*n* = 210)	Baseline GFAP level			*p value*
		Tertile 1 (*n* = 70)	Tertile 2 (*n* = 70)	Tertile 3 (*n* = 70)	
Age (y)	60.85 ± 9.78	57.05 ± 9.27	59.68 ± 10.16	65.82 ± 7.72	** < 0.001**
Gender (F/M)	74/136	32/38	24/46	18/52	**0.046**
Duration (m)	7.41 ± 6.97	8.59 ± 7.48	6.89 ± 6.70	6.74 ± 6.64	0.105
Education (y)	16.12 ± 2.62	16.01 ± 2.58	16.00 ± 2.60	16.36 ± 2.70	0.692
Follow-up time (y)	6.42 ± 1.69	6.29 ± 1.93	6.51 ± 1.49	6.47 ± 1.65	0.555
APOE ɛ4 carriers (%)	53(27.2)	22(34.9)	14(20.9)	17(26.2)	0.199
Aβ42 (pg/ml)	887.04 ± 385.84	809.72 ± 318.93	905.25 ± 380.08	947.93 ± 442.90	0.198
T-tau (pg/ml)	162.90 ± 57.68	142.60 ± 53.14	155.87 ± 49.71	190.23 ± 59.64	**< 0.001**
P-tau (pg/ml)	13.74 ± 5.77	11.77 ± 5.17	12.99 ± 5.17	16.45 ± 5.96	**< 0.001**
α-syn (pg/ml)	1505.71 ± 704.95	1358.79 ± 670.37	1379.58 ± 521.35	1778.76 ± 818.77	**< 0.001**
GFAP (ng/ml)	6.32 ± 3.01	3.59 ± 0.69	5.63 ± 0.64	9.76 ± 2.55	**< 0.001**
MoCA	27.18 ± 1.98	27.19 ± 2.03	27.30 ± 1.93	27.06 ± 1.99	0.770
HVLT Total Recall	46.30 ± 10.29	45.41 ± 10.77	45.60 ± 10.15	47.89 ± 9.89	0.263
HVLT Delayed Recall	45.63 ± 10.68	45.51 ± 10.91	43.44 ± 11.40	47.93 ± 9.28	**0.033**
HVLT Retention	47.75 ± 11.16	47.39 ± 10.52	45.84 ± 12.37	50.03 ± 10.21	0.057
HVLT Recognition Discrimination	45.59 ± 11.34	44.60 ± 12.70	45.66 ± 11.17	46.54 ± 10.05	0.428
JoLO	13.17 ± 1.83	13.10 ± 1.79	13.26 ± 1.79	13.14 ± 1.94	0.871
LNS	10.75 ± 2.55	11.41 ± 2.70	10.64 ± 2.27	10.19 ± 2.56	0.055
Semantic Fluency Test	50.69 ± 12.01	52.40 ± 13.58	51.19 ± 10.82	48.47 ± 11.29	0.160
SDMT	42.30 ± 8.94	43.26 ± 8.57	43.61 ± 10.02	40.03 ± 7.79	0.061

Categorical variables are reported as numbers and percentages; continuous variables are reported as means ± standard deviations

*P*values were assessed by ANOVA and Chi-square tests among the tertiles group

The bold emphasis in the table means *p* < 0.05

*CSF* cerebrospinal fluid, *PD* Parkinson’s disease, *APOE* Apolipoprotein E, *Aβ42* Amyloid-β42, *T-tau* Total tau, *P-tau* Phosphorylated tau, *α-syn* α-synuclein, *GFAP* glial fibrillary acidic protein, *MoCA* Montreal Cognitive Assessment, *HVLT* Hopkins Verbal Learning Test, *JoLO* Benton Judgment of Line Orientation, *LNS* Letter Number Sequencing, *SDMT* Symbol Digit Modality Test

PD patients were stratified into three groups according to tertiles of baseline CSF GFAP concentrations, which exhibited analogous baseline characteristics with respect to disease duration, education level, APOE ε4 allele status, and performance on global cognitive assessment. Notably, the levels of CSF biomarkers associated with neurodegeneration varied significantly among the groups (T-tau, P-tau, α-syn, all *p* < 0.001), except for Aβ42. In addition, statistically significant variations in age (*p* < 0.001) and gender (*p* = 0.046) were observed among the three groups.

**Fig. 1:**
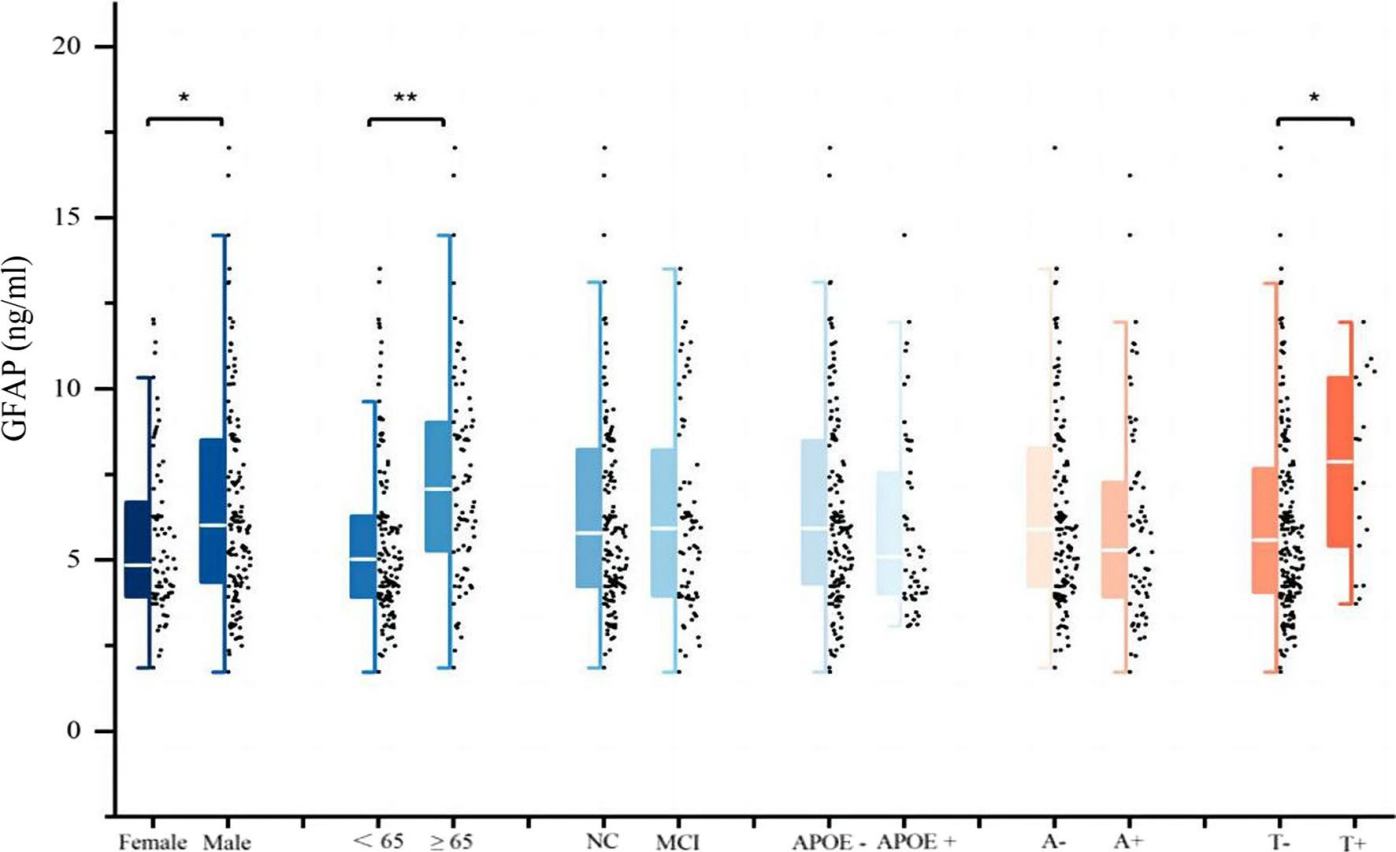
Baseline CSF GFAP levels of patients with de novo PD in different diagnostic groups. *p* values were assessed by Student’s *t* test between two groups. *p < 0.05, **p < 0.001. *CSF* cerebrospinal fluid, *GFAP* glial fibrillary acidic protein, *PD* Parkinson's disease, *NC* normal cognition, *MCI* mild cognitive impairment, *APOE* Apolipoprotein E, *A* Amyloid, *T* tau

In the PD cohort, CSF GFAP exhibited a statistically significant increase in the elderly population compared to the middle-aged population (*p* < 0.001) (Fig. 1). Furthermore, the multiple linear regression analysis demonstrated a positive correlation between CSF GFAP and age (β = 0.918, *p* < 0.001) (Additional file 1: Fig. S2). Analogously, the CSF GFAP concentrations of male participants surpassed those of female participants (*p* = 0.025), while no difference in CSF GFAP was observed between APOE ε4 carrier status (*p* = 0.384) (Fig. 1).

### Baseline associations of CSF GFAP with CSF biomarkers and cognitive functions

To evaluate the alterations in CSF GFAP throughout the pathology of PD, the whole cohort was partitioned into two groups based on CSF Aβ42 (A + and A −) or CSF P-tau (T + and T −). The T + group displayed higher levels of CSF GFAP compared to the T − group (*p* = 0.024), while no significant difference was observed in CSF GFAP levels between the A + and A − groups (*p* = 0.205) (Fig. 1). Following adjustment for confounding variables such as age, sex, education level, APOE ε4 carrier status and PD duration, the study noted that CSF GFAP had cross-sectional correlations with CSF Aβ42 (β = 0.162, *p* = 0.035), T-tau (β = 0.337, *p* < 0.001), P-tau (β = 0.375, *p* < 0.001), and α-syn (β = 0.311, *p* < 0.001) (Additional file 1: Table S2). Furthermore, mediation analysis revealed that at baseline levels, CSF GFAP mediated the effect of CSF Aβ42 on T-tau/P-tau by 7.12% and 6.56%, respectively (Additional file 1: Fig. S3a, b).

Our investigation revealed that there were no notable variances in baseline CSF GFAP levels between PD–NC and PD–MCI (*p* = 0.874) (Fig. 1). Moreover, the multiple linear regression analysis showed that CSF GFAP level was not associated with cognitive severity (assessed by MoCA total score) in the PD cohort at baseline (β = − 0.005, *p* = 0.721) (Additional file 1: Table S2).

### Prediction of longitudinal changes of CSF biomarkers and cognitive decline using baseline CSF GFAP

**Fig. 2:**
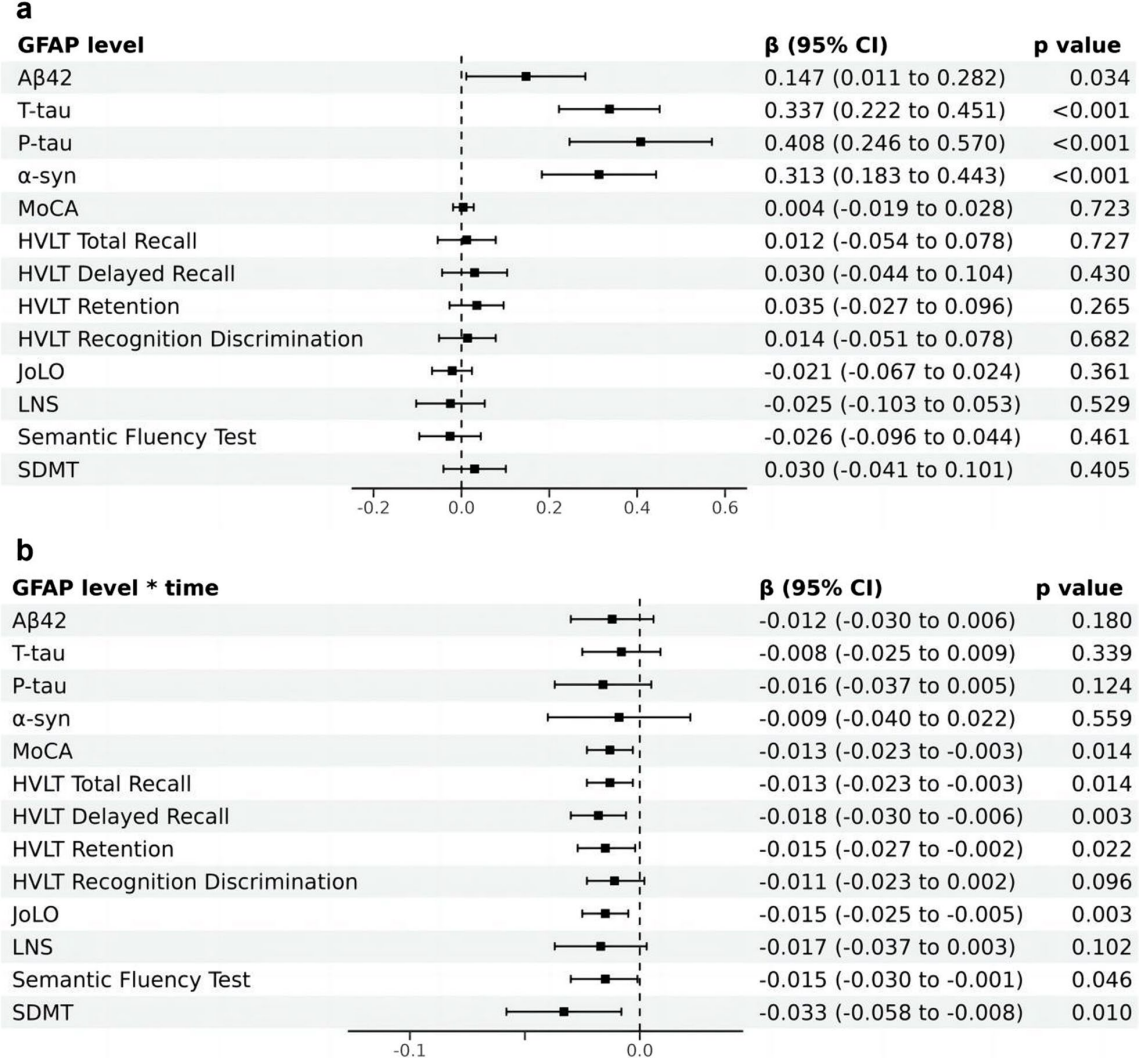
Effects of baseline GFAP **(a)** and GFAP*time **(b)** on the longitudinal alterations of other indicators. The regression coefficients (β) and adjusted *p* values were assessed by generalized linear mixed models. Adjusted for age, gender, educated level, APOE ε4 carrier status and duration. *GFAP* glial fibrillary acidic protein, *95% CI* 95% confidence interval, *Aβ42* Amyloid-β 42, *T-tau* Total tau, *P-tau* Phosphorylated tau, *α-syn* α-synuclein, *MoCA* Montreal Cognitive Assessment, *HVLT* Hopkins Verbal Learning Test, *JoLO* Benton Judgment of Line Orientation, *LNS* Letter Number Sequencing, *SDMT* Symbol Digit Modality Test

In de novo PD patients, baseline CSF GFAP levels also predicted the longitudinal increase of CSF Aβ42 (β = 0.147, *p* = 0.034), T-tau (β = 0.337, *p* < 0.001), and P-tau (β = 0.408, *p* < 0.001), α-syn (β = 0.313, *p* < 0.001) (Fig. 2a and Additional file 1: Table S3 Model 1). In addition, the associations were more pronounced among individuals with higher baseline CSF GFAP concentrations in the third tertile (T-tau, P-tau, α-syn, all *p* < 0.001) (Additional file 1: Table S4). Mediation analysis revealed that baseline CSF GFAP mediated the longitudinal effect of CSF Aβ42 on T-tau/P-tau by 8.37% and 7.41%, respectively (Additional file 1: Fig. S3c, d).

On the basis of prior research on biomarkers [16, 36, 37], we conducted an exploratory analysis to investigate the predictive value of CSF biomarkers in PD cognition. Hypothetically, upregulation of GFAP would be linked to cognitive impairment in several domains. The GLMM showed that higher baseline levels of CSF GFAP predicted a more rapid decline over time not only in global cognition (MoCA, β = − 0.013, *p* = 0.014), but also in episodic memory (HVLT Total Recall, β = − 0.013, *p* = 0.014; HVLT Delayed Recall, β = − 0.018, *p* = 0.003; HVLT Retention, β = − 0.015, *p* = 0.022), visuospatial abilities (JoLO, β = − 0.015, *p* = 0.003), language (Semantic Fluency Test, β = − 0.015, *p* = 0.046), as well as processing speed/attention (SDMT, β = − 0.033, *p* = 0.010) (Fig. 2b and Additional file 1: Table S5 Model 1). Furthermore, compared to the first and the second tertiles, higher levels of CSF GFAP in the third tertile had a significant predictive effect on the annual decline rate of PD cognition (Additional file 1: Fig. S4 and Table S6).

**Fig. 3:**
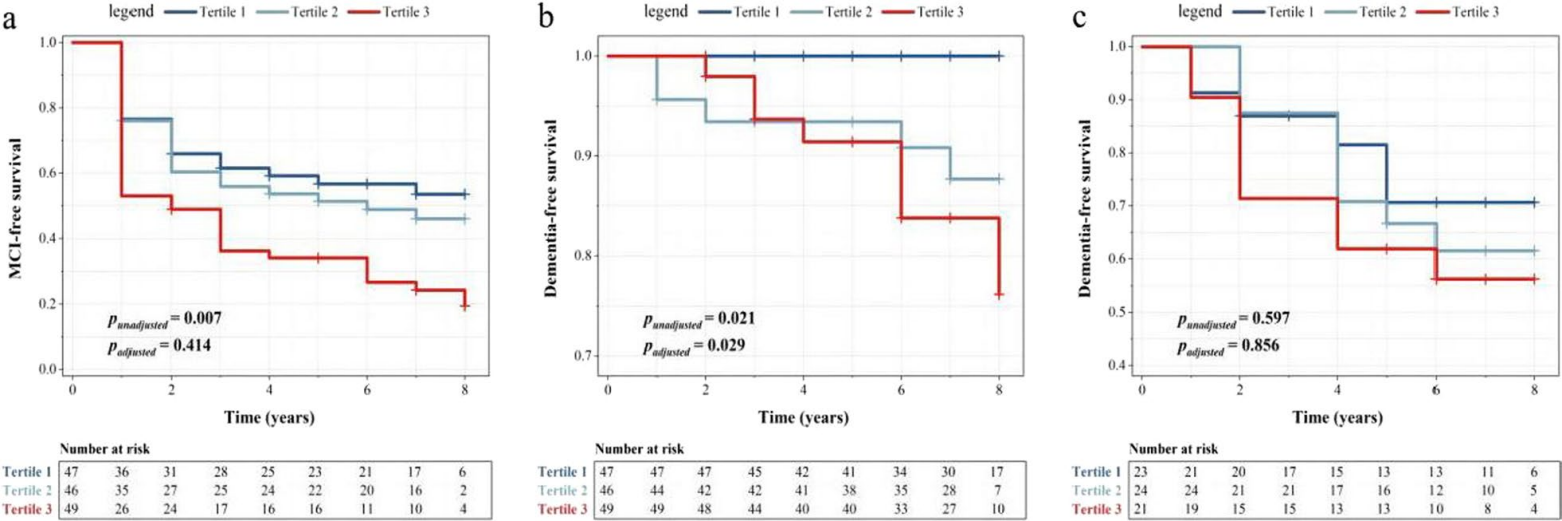
Cumulative probability risk of cognitive phase conversion in the follow-up among PD participants. **a** PD–NC conversion to PD–MCI,** b** PD–NC conversion to PD–D,** c** PD–MCI conversion to PD–D. Log-rank test: *P*_unadjusted_; Multivariate Cox regression analysis: *P*_adjusted_. *PD-NC* Parkinson's disease with normal cognition, *PD-MCI* Parkinson's disease with mild cognitive impairment, *PD-D* Parkinson's disease with dementia

To determine the potential of baseline CSF GFAP levels to serve as a predictor of cognitive transition (from NC to MCI or dementia, or from MCI to dementia) in patients with newly diagnosed PD over an 8-year follow-up period, we performed Kaplan–Meier survival curves and Cox regression analysis. Figure 3 shows the results of Kaplan–Meier analysis and log-rank test: in the PD–NC group (*n* = 142), the higher the baseline CSF GFAP level of the participants, the higher the risk of cognitive transition (MCI, *p* = 0.007; dementia, *p* = 0.021). However, in the PD–MCI group (*n* = 68), no significant predictive effect of CSF GFAP on the risk of dementia was found (*p* = 0.597). Meanwhile, multivariate Cox regression analysis revealed that higher baseline CSF GFAP levels in PD–NC patients could still predict the risk of dementia during 8 years of follow-up, even after controlling for age, gender, education level, APOE ε4 carrying status and disease duration (HR = 3.070, 95% CI 1.119–8.418, *p* = 0.029; Additional file 1: Table S7 Model 1).

Finally, to exclude the influence of other CSF biomarkers on PD cognition, we included several extra confounding factors (α-syn, Aβ42, P-tau), yielding results similar to those presented above (Additional file 1: Tables S3, S5 and S7 Model 2).

### Subgroup analysis

The independent predictive value of CSF GFAP for longitudinal increases in CSF Aβ42, T-tau, P-tau and α-syn did not vary significantly with gender, age, clinical cognitive diagnosis, and APOE ε4 allele carrier (Additional file 1: Tables S8–S11). However, in the Aβ-positive PD group, the association between baseline CSF GFAP and longitudinal CSF T-tau, P-tau, α-syn changes appeared to be more pronounced (Additional file 1: Table S12). Similarly, in male PD group, the baseline GFAP interaction with time seemed to have a more remarkable and comprehensive predictive effect on cognitive decline, mainly in episodic memory, visuospatial ability, language and processing speed/attention (Additional file 1: Table S8).

## Discussion

In the present study, we examined the cross-sectional and longitudinal associations of CSF GFAP with cognitive decline and changes in CSF biomarkers. Preliminary findings revealed that PD patients who had greater baseline CSF GFAP levels experienced more rapid cognitive deterioration during follow-up, indicating that CSF GFAP may be a predictor of cognitive impairment progression. Meanwhile, CSF GFAP was observed to be associated with AD-related pathological markers.

GFAP, an essential cytoskeletal component, is specifically expressed in astrocytes [38]. In addition, GFAP is mainly expressed in the hippocampus, corpus callosum and cerebral peduncle, with the highest expression levels observed in the hippocampus [39]. However, under pathological conditions, increased GFAP expression reflects astrocyte activation and is believed to be a pivotal contributor to the pathogenesis of neurodegenerative diseases, such as AD and PD [14]. An et al. demonstrated that GFAP adenovirus-induced reactive astrocyte proliferation exacerbated A53T-α-syn-mediated PD pathology [40]. Similarly, Herrera et al. reported an increase in GFAP-positive staining area and a decrease in synaptic transmission in the hippocampus of 6-hydroxydopamine model mice, accompanied by deficits in memory processing, indicating that astrocytes may adversely affect hippocampal function and cognitive behavior [41]. These studies suggest a close association between GFAP and the progression of PD. Consequently, GFAP may be considered a promising candidate biomarker for PD. Two case–control studies have found that CSF GFAP concentrations increase in both PD and PD–D patients compared with controls [36, 37].

In the newly diagnosed untreated PD cohort, we noted a discernible dissimilarity in CSF GFAP concentrations between male and female patients. This observation is in line with the work of Oeckl et al., who demonstrated that gender significantly influenced both serum and CSF GFAP levels in patients with cognitively preserved PD, dementia with Lewy bodies, PD–D, and behavioral variant frontotemporal dementia [36]. Moreover, we uncovered a noteworthy affirmative association between age and CSF GFAP concentrations in individuals with PD. However, Oeckl failed to find a correlation between GFAP and age in patients with the aforementioned degenerative diseases, and only detected a moderate correlation between serum GFAP and age in the control group (non-neurodegenerative disease) [36]. These results are incongruous with our own research findings.

In this study, we grouped participants according to their baseline cognitive level and found no difference in baseline CSF GFAP concentrations between the PD–NC and PD–MCI groups. Correspondingly, a prior study has illustrated that there exists no statistical difference in CSF GFAP concentrations between the PD–NC and PD–D groups [36]. This indicates that CSF GFAP cannot distinguish PD–NC from PD accompanied by cognitive dysfunction, and hence cannot be considered as a definitive diagnostic biomarker for PD–MCI patients.

Abnormal deposits of α-syn, tau and Aβ are seen in the brains of PD–D patients [42]. Meanwhile, AD neuropathological changes are considered to be an important determinant of PD–D [43]. We applied P-tau and Aβ42 as cutoff points to analyze AD-associated CSF profiles of enrolled PD individuals. PD patients in the T + group had higher GFAP concentrations than those in the T − group, meaning that elevation of GFAP in CSF was correlated with tau pathology. However, we found that Aβ pathology did not affect CSF GFAP levels. An AD-related study from the Translational Biomarkers in Aging and Dementia (TRIAD) cohort reported that CSF GFAP levels were notably elevated in both the A + T − and A + T + groups when compared to the A–T − group, although no statistical differences were discerned between the A + T − and A + T + groups [44]. Thus, our study suggests that the distribution of CSF GFAP in PD is distinct from that in aging and AD.

In light of key pathophysiological mechanisms, CSF α-syn, tau, and Aβ42 are considered as candidate biomarkers for assessing the progression of PD [25]. CSF levels of α-syn, T-tau, and P-tau increased over time in PD patients, and an increase in P-tau was linked to a swifter onset of motor symptoms and cognitive deterioration [45]. In addition, longitudinal alterations in CSF Aβ42 were observed to have a significant correlation with the extent of dopaminergic neuron loss in the left caudate nucleus [46]. Accordingly, comprehending the longitudinal changes of CSF biomarkers is helpful to monitor the severity and progress of clinical symptoms in PD patients. Bartl's study indicated that baseline CSF GFAP was positively associated with baseline CSF α-syn, T-tau, P-tau, and Aβ42, suggesting that GFAP can be used as a predictor for examining pathological changes occurring in PD [16]. However, the potential of CSF GFAP levels to predict longitudinal changes in these CSF biomarkers has not been systematically studied in other cohorts. Our results showed that baseline CSF GFAP was associated with longitudinal accumulation of CSF α-syn, T-tau, P-tau and Aβ42 pathology. Thus, GFAP may serve as a predictor of neuronal degeneration progression in patients with PD. Johansson et al. also proposed that GFAP might reflect AD pathology upstream to accumulation of tau protein tangles and neurodegeneration [47]. However, the positive correlation observed between GFAP and Aβ42 in CSF in our study poses a perplexing issue. Although this result is partially supported by Bartl's study [16], the exact mechanism remains unclear and further studies are, therefore, required. Furthermore, to our knowledge, Qin et al. reached similar results in their analysis of the PPMI cohort: there was also a significant positive correlation between the soluble fragment of triggering receptor expressed on myeloid cells 2 (sTREM2, a biomarker of microglial activation) and Aβ42 in CSF, both cross-sectionally and longitudinally [48]. In addition, through a subgroup analysis, our investigation revealed that the intensity of the correlation existing between baseline CSF GFAP and longitudinal CSF T-tau, P-tau, α-syn changes was influenced by Aβ status. In this regard, the study conducted by Benedet et al. in AD demonstrated that the connection between plasma GFAP concentrations and tau biomarkers was regulated by the Aβ status through mediation analysis [23]. Hence, a comprehensive examination of the function of GFAP necessitates the consideration of the impact of Aβ.

Bellaver et al. confirmed that in preclinical AD, high levels of plasma GFAP hold significance in the association between Aβ and early tau phosphorylation [24]. Similarly, Pereira et al. identified the potential mediating role of plasma GFAP in the association between Aβ PET and tau PET during the progression of AD [15]. In the present study, we determined that CSF GFAP acts as a partial mediator of the relationship between Aβ and T-tau or P-tau in newly diagnosed and untreated PD patients through mediation analysis. These findings suggest that there may be intricate interactions between these biomarkers and further exploration could enhance our comprehension of the pathological processes underlying PD, thereby facilitating the identification of crucial targets for intervention.

Recently, a study from Sweden's BioFINDER-2 cohort reported that higher baseline CSF GFAP was a significant predictor of longitudinal cognitive deterioration across the entire cohort, encompassing both the cognitively unimpaired group (CU Aβ- and CU Aβ +) and the cognitively impaired group (MCI and AD dementia) [15]. We found that baseline CSF GFAP can also predict longitudinal changes in various cognitive domains over time in patients with new-onset PD. Several studies have confirmed that cognitive decline in PD is associated with multiple clinical variables and CSF biomarkers. Older age and male are independently associated with future cognitive impairment in PD patients [49]. Poorer baseline cognitive performance predicts future cognitive decline in PD [20, 50]. PD patients carrying APOE ε4 have faster cognitive impairment and higher probability of progression to dementia [51–53]. The lower the baseline CSF Aβ42 level, the greater the likelihood of cognitive damage earlier in the disease process [21, 54]. Hereby, we chose to adjust for age, gender, baseline cognitive level, APOE ε4 carrying status and Aβ status for further investigation, and eventually found that there was still a predominant relationship between CSF GFAP and cognitive decline. In addition, we performed survival analyses which showed that the higher the baseline CSF GFAP level in PD–NC patients, the higher the risk of dementia. All these results manifest that astrocytes may play a role in promoting cognitive deterioration in PD. Wilson et al. used 11C-BU99008 PET molecular imaging to indirectly evaluate astrocyte pathology in PD patients and discovered that loss of astrocyte function in cortical areas was associated with poorer MoCA scores, indicating that astrocytes are important in contributing to the development of cognitive dysfunction in PD [55]. Consequently, therapies targeting neuroinflammation may at least partially improve cognitive symptoms.

Ferrari-Souza et al. demonstrated that CSF GFAP levels were influenced by Aβ pathology in aging and AD. Then, they discovered that CSF GFAP partially mediated the impacts of Aβ pathology on both hippocampal atrophy and cognitive deficits through mediation analyses [44]. Our research findings indicate that CSF GFAP levels in de novo PD patients can exert an influence on both the longitudinal changes of AD-associated pathology and the longitudinal decline rate of cognitive level. Thus, we provide further confirmation that neuroinflammation may have an impact on cognitive function of patients, in part by influencing AD-associated pathological changes in the pathogenesis of PD.

Certainly, an additional noteworthy aspect to be highlighted in this investigation is that through subgroup analysis considering age, gender, clinical cognitive diagnosis, APOE ε4 allele, and Aβ status, respectively, we discovered that the predictive value of GFAP baseline level on cognitive decline in PD was significant but there were subtle differences, which were manifested in different cognitive domains as well as cognitive severity. Further investigation is necessary to examine the correlations between these variables and CSF GFAP with longitudinal cognitive deterioration in new-onset PD patients.

Likewise, Bartl et al. also analyzed data from PPMI. Unfortunately, they did not confirm GFAP as a prognostic biomarker for future cognitive decline and CSF biomarker changes in PD [16]. The primary reason is that the research purposes are different. Our study aimed to determine the predictive value of GFAP for longitudinal cognitive deterioration and trends in CSF biomarkers, while Bartl assessed whether the biomarker panel (NTK) that has been validated in AD can be equally established in the PD cohort. Secondly, there are differences in subsequent data, variable selection and statistical methods. We collected full follow-up data on participants' cognitive assessment within 8 years from the PPMI cohort. For variable selection, in addition to GFAP, we also included the interaction of GFAP and time. Subgroup analyses were conducted to further investigate the longitudinal relationship between GFAP and cognitive decline using GLMM adjusted for age, sex, education, APOE ε4 carrier status, and disease duration.

In addition, Tang et al. conducted a longitudinal study on PD–MCI patients (*n* = 31) with a disease duration exceeding 1 year, for a period of 4.1 years. They discovered that baseline plasma GFAP concentrations were significantly higher in the MCI conversion group (PD patients who developed from MCI to dementia during the entire follow-up period) than in the MCI stable group (PD patients who did not develop from MCI to dementia during the entire follow-up period). Moreover, the receiver operating characteristic curve displayed that baseline plasma GFAP had a sensitivity of 90% and a specificity of 81% to distinguish MCI stable group from MCI converted group at the optimal cutoff value of 100.2 pg/ml. Taken together, these findings imply that plasma GFAP holds promise as a viable biomarker in prognosticating the advancement to dementia in PD–MCI patients [17]. In this study, we identify CSF GFAP as a prospective biomarker for forecasting the development of dementia in newly diagnosed untreated PD–NC patients over 8 years. Therefore, further exploration is needed regarding the potential value of GFAP.

However, a few limitations of our research should be noted. First, we only focused on examining the associations of baseline CSF GFAP with cognitive decrement and CSF biomarkers, without conducting a continuous longitudinal analysis of CSF GFAP. Therefore, it is essential to further ascertain the trajectory of GFAP throughout the progression of PD and its prospective clinical significance as a longitudinal monitoring tool. Second, this PD longitudinal cohort exhibited certain data gaps, particularly during subsequent follow-up periods, potentially exerting a nuanced influence on our findings. Third, owing to constraints imposed by the sample size, subgroup analysis pertaining to the tau state was not conducted. Besides, the current investigation presumed a linear association between GFAP and future cognitive decline, as well as alterations in AD-related pathological markers in individuals with newly diagnosed PD. However, in the event of a non-linear association, the study may incur a type II error.

## Conclusion

Our study unveils for the first time that CSF GFAP can predict AD-associated pathological changes and later cognitive decline and transformation in new-onset PD patients. The baseline value of GFAP may help to identify patients at risk of rapid cognitive decline in future clinical trials to reduce heterogeneity.

## Supplementary Information

**Additional file 1: Figure S1.** Quantile–Quantile plot of GFAP. **Figure S2.** CSF GFAP concentration was positively correlated with age. **Figure S3.** Relationships between Aβ42 and T-tau or P-tau were mediated by baseline CSF GFAP. **Figure S4.** Relationship between CSF GFAP and longitudinal cognitive decline. **Table S1.** Number of data-points of longitudinal CSF biomarkers and cognitive assessments. **Table S2.** Baseline correlation of GFAP with CSF biomarkers and cognitive assessments. **Table S3.** Effects of baseline GFAP (continuous value) on longitudinal CSF biomarkers and cognitive progression. **Table S4.** Effects of baseline GFAP on longitudinal CSF biomarkers and cognitive progression in the tertiles. **Table S5.** Effects of baseline GFAP*time (continuous value) on longitudinal CSF biomarkers and cognitive progression. **Table S6.** Effects of baseline GFAP*time on longitudinal CSF biomarkers and cognitive progression in the tertiles. **Table S7.** Progression risk from NC to MCI or dementia or from MCI to dementia. **Table S8.** Prediction of baseline GFAP and GFAP*time in male and female patients with de novo PD. **Table S9.** Prediction of baseline GFAP and GFAP*time in de novo PD patients aged < 56 and ≥ 65. **Table S10.** Prediction of baseline GFAP and GFAP*time in patients with PD–NC and PD–MCI. **Table S11.** Prediction of baseline GFAP and GFAP*time in new-onset PD patients carrying APOE ε4 or not. **Table S12.** Prediction of baseline GFAP and GFAP*time in patients with Amyloid-PD and Amyloid + PD.

## Data Availability

All data generated or analysed during this study are included in this published article and/or its additional information files.

## Abbreviations

PD: Parkinson’s disease
GFAP: Glial fibrillary acidic protein
AD: Alzheimer’s disease
CSF: Cerebrospinal fluid
Aβ: Amyloid-β
PET: Positron emission tomography
PPMI: Parkinson’s Progression Markers Initiative
APOE: Apolipoprotein E
GLMM: Generalized linear mixed model
PD–NC: Parkinson’s disease with normal cognition
PD–MCI: Parkinson’s disease with mild cognitive impairment
PD–D: Parkinson’s disease dementia
CNS: Central nervous system
MoCA: Montreal Cognitive Assessment
T-tau: Total tau
α-syn: α-Synuclein
P-tau: Phosphorylated tau
HVLT: Hopkins Verbal Learning Test
JoLO: Benton Judgment of Line Orientation
LNS: Letter Number Sequencing
SDMT: Symbol Digit Modalities Test
