# Supplementary material for: Cerebrospinal fluid GFAP is a predictive biomarker for conversion to dementia and Alzheimer’s disease-associated biomarkers alterations among de novo Parkinson’s disease patients: a prospective cohort study
Source: J Neuroinflammation. 2023 Jul 20;20:167. doi: 10.1186/s12974-023-02843-5 (PMC10357612; doi:10.1186/s12974-023-02843-5)
Supplement: Supplementary file 1 — Additional file 1: Figure S1. Quantile–Quantile plot of GFAP. Figure S2. CSF GFAP concentration was positively correlated with age. Figure S3. Relationships between Aβ42 and T-tau or P-tau were mediated by baseline CSF GFAP. Figure S4. Relationship between CSF GFAP and longitudinal cognitive decline. Table S1. Number of data-points of longitudinal CSF biomarkers and cognitive assessments. Table S2. Baseline correlation of GFAP with CSF biomarkers and cognitive assessments. Table S3. Effects of baseline GFAP (continuous value) on longitudinal CSF biomarkers and cognitive progression. Table S4. Effects of baseline GFAP on longitudinal CSF biomarkers and cognitive progression in the tertiles. Table S5. Effects of baseline GFAP*time (continuous value) on longitudinal CSF biomarkers and cognitive progression. Table S6. Effects of baseline GFAP*time on longitudinal CSF biomarkers and cognitive progression in the tertiles. Table S7. Progression risk from NC to MCI or dementia or from MCI to dementia. Table S8. Prediction of baseline GFAP and GFAP*time in male and female patients with de novo PD. Table S9. Prediction of baseline GFAP and GFAP*time in de novo PD patients aged < 56 and ≥ 65. Table S10. Prediction of baseline GFAP and GFAP*time in patients with PD–NC and PD–MCI. Table S11. Prediction of baseline GFAP and GFAP*time in new-onset PD patients carrying APOE ε4 or not. Table S12. Prediction of baseline GFAP and GFAP*time in patients with Amyloid-PD and Amyloid + PD. [file 12974_2023_2843_MOESM1_ESM.pdf]

Figure S1 The Quantile-Quantile plot of GFAP

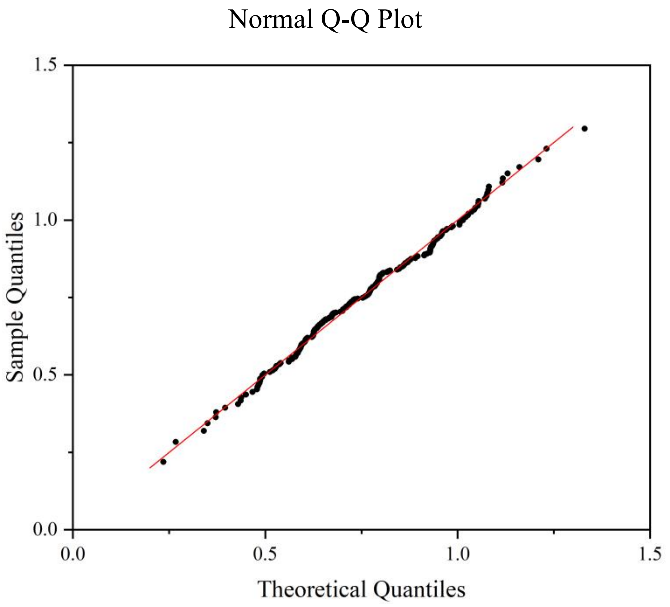

Sample quantiles were plotted follow those theoretical quantiles under the null hypothesis (x-axis). The Q-Q plot shows that log10-transformed CSF GFAP data conforms to the normal distribution. Abbreviations: CSF, cerebrospinal fluid; GFAP, glial fibrillary acidic protein.

Figure S2 CSF GFAP concentration was positively correlated with age

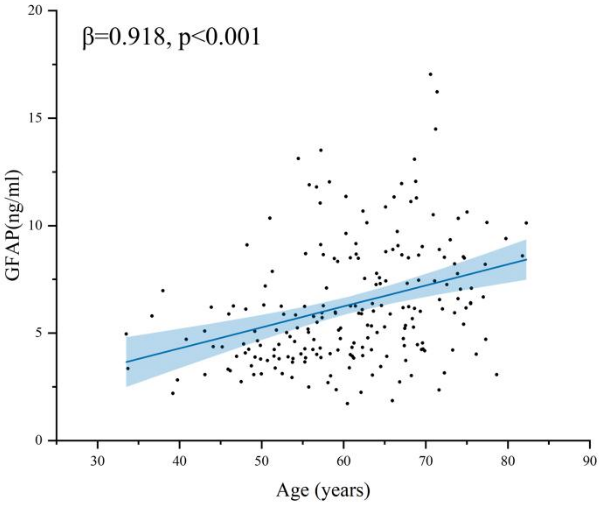

The regression coefficient (β) and p-value computed by multiple linear regression.

Abbreviations: CSF, cerebrospinal fluid; GFAP, glial fibrillary acidic protein.

Figure S3 The relationships between Aβ42 and T-tau or P-tau were mediated by baseline CSF GFAP

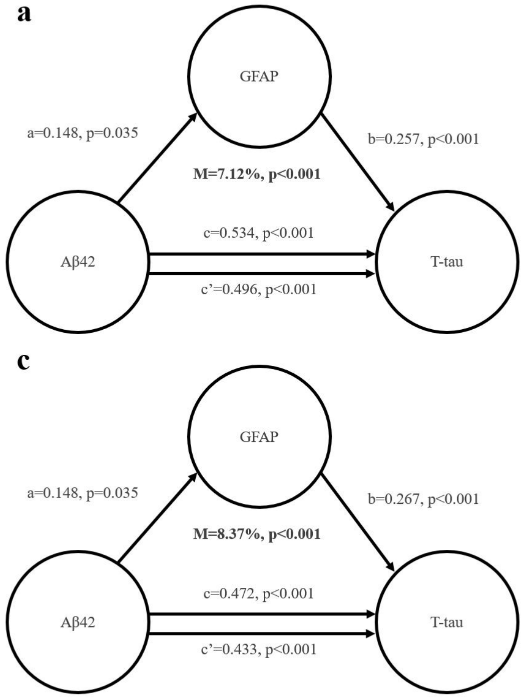

a b at beseline, **c d** during follow-up. Path a indicates the direct efect of Aβ42 on GFAP. Path b indicates the direct efect of GFAP on T-tau or P-tau. Path c' indicates the direct efect of Aβ42 on T-tau or P-tau. Path c indicates the total efect of Aβ42 on T-tau or P-tau. The regression coefficient and p-value computed by mediation analysis. Adjusted for age, gender, educational level, APOE ε4 carrier status, and disease duration.

Abbreviations: Aβ42, amyloid-β42; T-tau, total tau; P-tau, phosphorylated tau; CSF, cerebrospinal fluid; GFAP, glial fibrillary acidic protein; M, mediating effect.

Figure S4 Relationship between CSF GFAP and longitudinal cognitive decline

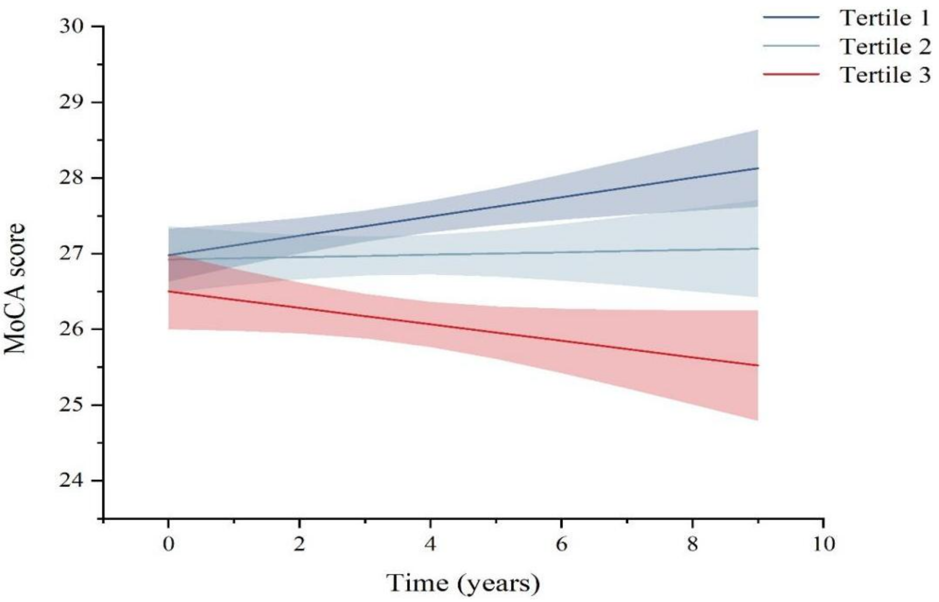

Abbreviations: CSF, Cerebrospinal fluid; GFAP, glial fibrillary acidic protein; MoCA, Montreal Cognitive Assessment.

Table S1 Number of data-points of longitudinal CSF biomarkers and cognitive assessments

Study visit	bl	m12	m24	m36	m48	m60	m72	m84	m96
Aβ42	205	196	197	161	106	90	32	43	
T-tau	210	199	200	168	106	90	32	43	
P-tau	210	199	200	168	106	90	32	43	
α-syn	210	198	200	146					
GFAP	210								
MoCA	210	206	205	191	180	166	151	132	62
HVLT Total Recall	210	206	206	193	181	166	150	132	61
HVLT Delayed Recall	210	206	206	192	181	166	150	132	61
HVLT Retention	209	204	206	191	181	166	150	132	61
HVLT Recognition Discrimination	210	206	206	192	181	166	149	132	61
JoLO	210	206	204	192	180	165	148	131	61
LNS	210	206	206	193	181	166			
Semantic Fluency Test	210	206	206	193	181	166			
SDMT	210	206	206	191	181	166			

Abbreviations: CSF, cerebrospinal fluid; Aβ42, amyloid-β42; T-tau, total tau; P-tau, phosphorylated tau; α-syn, α-synuclein; GFAP, glial fibrillary acidic protein; MoCA, Montreal Cognitive Assessment; HVLT, Hopkins Verbal Learning Test; JoLO, Benton Judgment of Line Orientation; LNS, Letter Number Sequencing; SDMT, Symbol Digit Modality Test.

Table S2 Baseline correlation of GFAP with CSF biomarkers and cognitive assessments

Baseline Measures	β	95% CI		*p value*
		lower	upper	
Aβ42	0.162	0.012	0.313	**0.035**
T-tau	0.337	0.209	0.466	**<0.001**
P-tau	0.375	0.213	0.536	**<0.001**
α-syn	0.311	0.167	0.455	**<0.001**
MoCA	0.005	-0.021	0.031	0.717
HVLT Total Recall	0.054	-0.027	0.136	0.189
HVLT Delayed Recall	0.107	0.016	0.198	**0.022**
HVLT Retention	0.135	0.040	0.230	**0.006**
HVLT Recognition Discrimination	0.092	-0.011	0.196	0.079
JoLO	-0.016	-0.068	0.035	0.539
LNS	-0.032	-0.119	0.054	0.461
Semantic Fluency Test	-0.022	-0.096	0.053	0.566
SDMT	0.007	-0.067	0.081	0.854

The regression coefficients (β) and adjusted p-values computed by multiple linear regression. Adjusted for age, gender, educational level, APOE ε4 carrier status, and disease duration.

The bold emphasis in the table means $p < 0.05$.

Abbreviations: CSF, cerebrospinal fluid; GFAP, glial fibrillary acidic protein; 95% CI, 95% confidence interval; Aβ42, amyloid-β 42; T-tau, total tau; P-tau, phosphorylated tau;α-syn, α-synuclein; MoCA, Montreal Cognitive Assessment; HVLT, Hopkins Verbal Learning Test; JoLO, Benton Judgment of Line Orientation; LNS, Letter Number Sequencing; SDMT, Symbol Digit Modality Test.

Table S3 Effects of baseline GFAP (continuous value) on longitudinal CSF biomarkers and cognitive progression

Follow-up measures	GFAP level							
	Model 1				Model 2			
	β	95% CI		*p value*	β	95% CI		*p value*
		lower	upper			lower	upper	
Aβ42	0.147	0.011	0.282	**0.034**	/	/	/	/
T-tau	0.337	0.222	0.451	**<0.001**	/	/	/	/
P-tau	0.408	0.246	0.570	**<0.001**	/	/	/	/
α-syn	0.313	0.183	0.443	**<0.001**	/	/	/	/
MoCA	0.004	-0.019	0.028	0.723	0.006	-0.019	0.032	0.617
HVLT Total Recall	0.012	-0.054	0.078	0.727	0.006	-0.064	0.076	0.867
HVLT Delayed Recall	0.030	-0.044	0.104	0.430	0.024	-0.054	0.103	0.542
HVLT Retention	0.035	-0.027	0.096	0.265	0.036	-0.030	0.102	0.283
HVLT Recognition Discrimination	0.014	-0.051	0.078	0.682	0.007	-0.062	0.075	0.849
JoLO	-0.021	-0.067	0.024	0.361	-0.030	-0.076	0.017	0.206
LNS	-0.025	-0.103	0.053	0.529	-0.048	-0.131	0.036	0.261
Semantic Fluency Test	-0.026	-0.096	0.044	0.461	-0.033	-0.108	0.042	0.386
SDMT	0.030	-0.041	0.101	0.405	0.001	-0.075	0.075	0.997

The regression coefficients (β) and adjusted p-values computed by generalized linear mixed models.

Model 1: adjusted for age, gender, educational level, APOE ε4 carrier status, and disease duration.

Model 2: adjusted for age, gender, educational level, APOE ε4 carrier status, disease duration, α-syn, Aβ42 and P-tau.

The bold emphasis in the table means $p < 0.05$.

Abbreviations: CSF, cerebrospinal fluid; GFAP, glial fibrillary acidic protein; 95% CI, 95% confidence interval; Aβ42, Amyloid-β 42; T-tau, Total tau; P-tau, Phosphorylated tau; α-syn, α-synuclein; MoCA, Montreal Cognitive Assessment; HVLT, Hopkins Verbal Learning Test; JoLO, Benton Judgment of Line Orientation; LNS, Letter Number Sequencing; SDMT, Symbol Digit Modality Test.

Table S4 Effects of baseline GFAP on longitudinal CSF biomarkers and cognitive progression in the tertiles

**Follow-up measures**	**GFAP level**								
	**Tertile 1**	**Tertile 2**				**Tertile 3**			
		**β**	**95% CI**		***p value***	**β**	**95% CI**		***p value***
			**lower**	**upper**			**lower**	**upper**	
Aβ42	Reference	0.035	-0.025	0.095	0.249	0.059	-0.006	0.123	0.077
T-tau	Reference	0.054	0.002	0.105	**0.041**	0.138	0.082	0.194	**<0.001**
P-tau	Reference	0.049	-0.023	0.121	0.182	0.179	0.101	0.257	**<0.001**
α-syn	Reference	0.035	-0.022	0.093	0.228	0.137	0.075	0.200	**<0.001**
MoCA	Reference	0.003	-0.008	0.013	0.595	0.004	-0.007	0.016	0.476
HVLT Total Recall	Reference	0.003	-0.026	0.033	0.815	0.004	-0.028	0.036	0.808
HVLT Delayed Recall	Reference	-0.005	-0.038	0.027	0.743	0.018	-0.018	0.053	0.325
HVLT Retention	Reference	-0.006	-0.033	0.021	0.652	0.020	-0.009	0.050	0.177
HVLT Recognition Discrimination	Reference	-0.004	-0.033	0.024	0.769	0.011	-0.020	0.042	0.479
JoLO	Reference	-0.005	-0.025	0.015	0.622	-0.013	-0.034	0.009	0.257
LNS	Reference	-0.012	-0.046	0.023	0.510	-0.004	-0.041	0.033	0.822
Semantic Fluency Test	Reference	0.006	-0.025	0.036	0.726	-0.009	-0.043	0.024	0.587
SDMT	Reference	0.010	-0.021	0.042	0.513	0.004	-0.020	0.048	0.421

The regression coefficients (β) and adjusted p-values computed by generalized linear mixed models.

Adjusted for age, gender, educational level, APOE ε4 carrier status, and disease duration.

The bold emphasis in the table means $p < 0.05$.

Abbreviations: CSF, cerebrospinal fluid; GFAP, glial fibrillary acidic protein; 95% CI, 95% confidence interval; Aβ42, Amyloid-β 42; T-tau, Total tau; P-tau, Phosphorylated tau; α-syn, α-synuclein; MoCA, Montreal Cognitive Assessment; HVLT, Hopkins Verbal Learning Test; JoLO, Benton Judgment of Line Orientation; LNS, Letter Number Sequencing; SDMT, Symbol Digit Modality Test.

Table S5 Effects of baseline GFAP*time (continuous value) on longitudinal CSF biomarkers and cognitive progression

Follow-up measures	GFAP level*time							
	Model 1				Model 2			
	β	95% CI		*p value*	β	95% CI		*p value*
		lower	upper			lower	upper	
Aβ42	-0.012	-0.030	0.006	0.180	/	/	/	/
T-tau	-0.008	-0.025	0.009	0.339	/	/	/	/
P-tau	-0.016	-0.037	0.005	0.124	/	/	/	/
α-syn	-0.009	-0.040	0.022	0.559	/	/	/	/
MoCA	-0.013	-0.023	-0.003	**0.014**	-0.014	-0.024	-0.003	**0.011**
HVLT Total Recall	-0.013	-0.023	-0.003	**0.014**	-0.010	-0.020	0.001	0.084
HVLT Delayed Recall	-0.018	-0.030	-0.006	**0.003**	-0.014	-0.026	-0.002	**0.026**
HVLT Retention	-0.015	-0.027	-0.002	**0.022**	-0.013	-0.026	0.001	0.055
HVLT Recognition Discrimination	-0.011	-0.023	0.002	0.096	-0.003	-0.016	0.010	0.701
JoLO	-0.015	-0.025	-0.005	**0.003**	-0.013	-0.023	-0.002	**0.019**
LNS	-0.017	-0.037	0.003	0.102	-0.017	-0.038	0.005	0.127
Semantic Fluency Test	-0.015	-0.030	-0.001	**0.046**	-0.014	-0.030	0.001	0.077
SDMT	-0.033	-0.058	-0.008	**0.010**	-0.028	-0.054	-0.002	**0.034**

The regression coefficients (β) and adjusted p-values computed by generalized linear mixed models.

Model 1: adjusted for age, gender, educational level, APOE ε4 carrier status, and disease duration.

Model 2: adjusted for age, gender, educational level, APOE ε4 carrier status, disease duration, α-syn, Aβ42 and P-tau.

The bold emphasis in the table means $p < 0.05$.

Abbreviations: CSF, cerebrospinal fluid; GFAP, glial fibrillary acidic protein; 95% CI, 95% confidence interval; Aβ42, Amyloid-β 42; T-tau, Total tau; P-tau, Phosphorylated tau; α-syn, α-synuclein; MoCA, Montreal Cognitive Assessment; HVLT, Hopkins Verbal Learning Test; JoLO, Benton Judgment of Line Orientation; LNS, Letter Number Sequencing; SDMT, Symbol Digit Modality Test.

Table S6 Effects of baseline GFAP*time on longitudinal CSF biomarkers and cognitive progression in the tertiles

Follow-up measures	GFAP level*time								
	Tertile 1	Tertile 2				Tertile 3			
		β	95% CI		*p value*	β	95% CI		*p value*
			lower	upper			lower	upper	
Aβ42	Reference	-0.002	-0.010	0.007	0.658	-0.006	-0.014	0.003	0.191
T-tau	Reference	0.004	-0.008	0.008	0.955	-0.002	-0.010	0.007	0.707
P-tau	Reference	0.002	-0.008	0.012	0.672	-0.007	-0.017	0.003	0.160
α-syn	Reference	0.001	-0.013	0.016	0.845	-0.002	-0.017	0.012	0.772
MoCA	Reference	-0.003	-0.008	0.001	0.167	-0.007	-0.011	-0.002	**0.007**
HVLT Total Recall	Reference	0.002	-0.003	0.007	0.483	-0.006	-0.011	-0.001	**0.020**
HVLT Delayed Recall	Reference	0.001	-0.005	0.007	0.747	-0.008	-0.013	-0.002	**0.009**
HVLT Retention	Reference	0.001	-0.006	0.007	0.858	-0.005	-0.012	0.001	0.079
HVLT Recognition Discrimination	Reference	-0.004	-0.010	0.002	0.232	-0.008	-0.014	-0.001	**0.015**
JoLO	Reference	-0.003	-0.007	0.002	0.291	-0.008	-0.013	-0.003	**0.001**
LNS	Reference	-0.002	-0.011	0.007	0.670	-0.011	-0.020	-0.001	**0.024**
Semantic Fluency Test	Reference	-0.001	-0.007	0.007	0.937	-0.009	-0.016	-0.002	**0.012**
SDMT	Reference	-0.002	-0.014	0.010	0.706	-0.017	-0.029	-0.005	**0.005**

The regression coefficients (β) and adjusted p-values computed by generalized linear mixed models.

Adjusted for age, gender, educational level, APOE ε4 carrier status, and disease duration.

The bold emphasis in the table means $p < 0.05$.

Abbreviations: CSF, cerebrospinal fluid; GFAP, glial fibrillary acidic protein; 95% CI, 95% confidence interval; Aβ42, Amyloid-β 42; T-tau, Total tau; P-tau, Phosphorylated tau; α-syn, α-synuclein; MoCA, Montreal Cognitive Assessment; HVLT, Hopkins Verbal Learning Test; JoLO, Benton Judgment of Line Orientation; LNS, Letter Number Sequencing; SDMT, Symbol Digit Modality Test.

Table S7 Progression risk from NC to MCI or dementia or from MCI to dementia

Cognition progression	HR	95% CI		*p value*
		lower	upper	
**Model 1**				
PD-NC convert to PD-MCI	1.145	0.828	1.583	0.414
PD-NC convert to PD-D	3.070	1.119	8.418	**0.029**
PD-MCI convert to PD-D	1.052	0.607	1.826	0.856
**Model 2**				
PD-NC convert to PD-MCI	1.198	0.827	1.736	0.339
PD-NC convert to PD-D	4.265	1.240	14.669	**0.021**
PD-MCI convert to PD-D	1.217	0.677	2.189	0.512

The HR and adjusted p-values computed by multivariate cox regression models.

Model 1: adjusted for age, gender, educational level, APOE ε4 carrier status, and disease duration.

Model 2: adjusted for age, gender, educational level, APOE ε4 carrier status, disease duration, α-syn, Aβ42 and P-tau.

The bold emphasis in the table means $p < 0.05$.

Abbreviations: PD-NC, Parkinson's disease with normal cognition; PD-MCI, Parkinson's disease with mild cognitive impairment; PD-D, Parkinson's disease with dementia; HR, hazard ratio; 95% CI, 95% confidence interval.

Table S8 Prediction of baseline GFAP and GFAP*time in male and female patients with de novo PD

Measures	GFAP level				GFAP level*time			
	Female		Male		Female		Male	
	**β**	***p***	**β**	***p***	**β**	***p***	**β**	***p***
Aβ42	0.106	0.409	**0.182**	**0.036**	-0.024	0.178	-0.007	0.490
T-tau	**0.247**	**0.009**	**0.363**	**<0.001**	-0.004	0.847	-0.009	0.371
P-tau	**0.304**	**0.015**	**0.435**	**<0.001**	-0.026	0.246	-0.016	0.203
α-syn	**0.246**	**0.043**	**0.323**	**<0.001**	-0.012	0.718	-0.011	0.540
MoCA	0.001	0.957	-0.004	0.795	-0.005	0.205	-0.012	0.075
HVLT Total Recall	0.017	0.806	0.006	0.885	0.003	0.722	**-0.020**	**0.002**
HVLT Delayed Recall	0.006	0.937	0.040	0.394	-0.003	0.807	**-0.026**	**0.001**
HVLT Retention	0.005	0.930	0.039	0.311	-0.004	0.728	**-0.020**	**0.012**
HVLT Recognition Discrimination	-0.001	0.982	0.021	0.600	-0.003	0.767	**-0.017**	**0.034**
JoLO	-0.015	0.774	-0.015	0.555	-0.010	0.144	**-0.014**	**0.030**
LNS	0.016	0.848	-0.053	0.251	0.004	0.825	-0.024	0.060
Semantic Fluency Test	-0.124	0.079	0.012	0.775	0.012	0.380	**-0.025**	**0.010**
SDMT	0.003	0.966	0.035	0.449	-0.019	0.496	**-0.042**	**0.005**

The regression coefficients (β) and adjusted p-values computed by generalized linear mixed models.

Adjusted for age, gender, educational level, APOE ε4 carrier status, and disease duration.

The bold emphasis in the table means $p < 0.05$.

Abbreviations: GFAP, glial fibrillary acidic protein; PD, Parkinson's disease; Aβ42, Amyloid-β 42; T-tau, Total tau; P-tau, Phosphorylated tau; α-syn, α-synuclein; MoCA, Montreal Cognitive Assessment; HVLT, Hopkins Verbal Learning Test; JoLO, Benton Judgment of Line Orientation; LNS, Letter Number Sequencing; SDMT, Symbol Digit Modality Test.

Table S9 Prediction of baseline GFAP and GFAP*time in de novo PD patients aged <56 and≥65

Measures	GFAP level				GFAP level*time			
	< 65 years		⩾ 65 years		< 65 years		⩾ 65 years	
	**β**	***p***	**β**	***p***	**β**	***p***	**β**	***p***
Aβ42	0.120	0.145	0.215	0.083	-0.002	0.888	**-0.046**	**0.008**
T-tau	**0.364**	**<0.001**	**0.273**	**0.004**	-0.008	0.508	**-0.036**	**0.006**
P-tau	**0.431**	**<0.001**	**0.355**	**0.011**	-0.022	0.168	**-0.036**	**0.030**
α-syn	**0.312**	**<0.001**	**0.336**	**0.003**	-0.007	0.751	-0.002	0.951
MoCA	0.010	0.488	-0.006	0.791	**-0.016**	**0.001**	-0.001	0.999
HVLT Total Recall	-0.007	0.870	0.033	0.502	-0.013	0.104	-0.008	0.289
HVLT Delayed Recall	0.002	0.972	0.058	0.340	-0.014	0.110	-0.016	0.083
HVLT Retention	0.008	0.837	0.065	0.242	-0.007	0.449	-0.017	0.101
HVLT Recognition Discrimination	-0.020	0.615	0.058	0.310	-0.008	0.337	-0.006	0.528
JoLO	-0.032	0.276	-0.016	0.670	**-0.021**	**0.003**	-0.007	0.274
LNS	-0.039	0.454	0.016	0.797	-0.010	0.474	-0.014	0.449
Semantic Fluency Test	-0.036	0.405	-0.006	0.922	-0.009	0.255	-0.016	0.359
SDMT	0.007	0.883	0.072	0.230	-0.024	0.069	-0.033	0.279

The regression coefficients (β) and adjusted p-values computed by generalized linear mixed models.

Adjusted for age, gender, educational level, APOE ε4 carrier status, and disease duration.

The bold emphasis in the table means $p < 0.05$.

Abbreviations: GFAP, glial fibrillary acidic protein; PD, Parkinson's disease; Aβ42, Amyloid-β 42; T-tau, Total tau; P-tau, Phosphorylated tau; α-syn, α-synuclein; MoCA, Montreal Cognitive Assessment; HVLT, Hopkins Verbal Learning Test; JoLO, Benton Judgment of Line Orientation; LNS, Letter Number Sequencing; SDMT, Symbol Digit Modality Test.

Table S10 Prediction of baseline GFAP and GFAP*time in patients with PD-NC and PD-MCI

Measures	GFAP level				GFAP level*time			
	NC		MCI		NC		MCI	
	**β**	***p***	**β**	***p***	**β**	***p***	**β**	***p***
Aβ42	0.102	0.218	0.228	0.066	-0.007	0.502	-0.028	0.108
T-tau	**0.340**	**<0.001**	**0.368**	**0.001**	0.003	0.751	**-0.039**	**0.020**
P-tau	**0.419**	**<0.001**	**0.416**	**0.009**	-0.010	0.394	-0.037	0.079
α-syn	**0.310**	**<0.001**	**0.325**	**0.011**	-0.018	0.340	0.009	0.771
MoCA	0.004	0.738	-0.008	0.698	-0.008	0.135	**-0.022**	**0.026**
HVLT Total Recall	0.017	0.663	-0.014	0.797	**-0.019**	**0.005**	-0.002	0.811
HVLT Delayed Recall	0.015	0.753	0.025	0.679	**-0.018**	**0.011**	-0.016	0.137
HVLT Retention	0.013	0.733	0.052	0.317	-0.010	0.202	-0.023	0.058
HVLT Recognition Discrimination	-0.014	0.719	0.036	0.525	**-0.017**	**0.023**	0.003	0.794
JoLO	-0.040	0.131	0.017	0.701	-0.006	0.182	**-0.031**	**0.012**
LNS	-0.055	0.210	0.023	0.769	-0.011	0.249	-0.028	0.239
Semantic Fluency Test	-0.030	0.456	-0.021	0.717	-0.002	0.823	**-0.040**	**0.009**
SDMT	0.038	0.371	0.019	0.786	-0.013	0.130	-0.068	0.052

The regression coefficients (β) and adjusted p-values computed by generalized linear mixed models.

Adjusted for age, gender, educational level, APOE ε4 carrier status, and disease duration.

The bold emphasis in the table means $p < 0.05$.

Abbreviations: GFAP, glial fibrillary acidic protein; PD-NC, Parkinson's disease with normal cognition; PD-MCI, Parkinson's disease with mild cognitive impairment; Aβ42, Amyloid-β 42; T-tau, Total tau; P-tau, Phosphorylated tau; α-syn, α-synuclein; MoCA, Montreal Cognitive Assessment; HVLT, Hopkins Verbal Learning Test; JoLO, Benton Judgment of Line Orientation; LNS, Letter Number Sequencing; SDMT, Symbol Digit Modality Test.

Table S11 Prediction of baseline GFAP and GFAP*time in new-onset PD patients carrying APOE ε4 or not

Measures	GFAP level				GFAP level*time			
	APOE ε4 -		APOE ε4 +		APOE ε4 -		APOE ε4 +	
	β	*p*	β	*p*	β	*p*	β	*p*
Aβ42	**0.206**	**0.006**	-0.128	0.437	-0.015	0.125	-0.014	0.523
T-tau	**0.304**	**<0.001**	**0.429**	**0.001**	-0.011	0.277	0.010	0.460
P-tau	**0.341**	**<0.001**	**0.629**	**0.004**	-0.013	0.271	-0.033	0.209
α-syn	**0.280**	**<0.001**	**0.439**	**0.014**	-0.005	0.781	-0.022	0.407
MoCA	0.005	0.733	-0.043	0.269	**-0.010**	**0.010**	-0.027	0.109
HVLT Total Recall	0.011	0.751	0.050	0.604	-0.008	0.171	**-0.035**	**0.011**
HVLT Delayed Recall	0.036	0.383	-0.013	0.906	**-0.016**	**0.016**	-0.024	0.103
HVLT Retention	0.047	0.175	-0.054	0.526	**-0.015**	**0.038**	-0.015	0.306
HVLT Recognition Discrimination	0.011	0.760	0.082	0.361	-0.001	0.892	**-0.052**	**0.001**
JoLO	-0.006	0.804	-0.068	0.309	-0.010	0.060	**-0.034**	**0.003**
LNS	-0.039	0.304	-0.025	0.838	-0.008	0.459	**-0.057**	**0.034**
Semantic Fluency Test	-0.040	0.303	0.057	0.551	-0.003	0.638	**-0.060**	**0.006**
SDMT	0.010	0.779	0.147	0.226	-0.016	0.190	**-0.096**	**0.005**

The regression coefficients (β) and adjusted p-values computed by generalized linear mixed models.

Adjusted for age, gender, educational level, APOE ε4 carrier status, and disease duration.

The bold emphasis in the table means $p < 0.05$.

Abbreviations: GFAP, glial fibrillary acidic protein; PD, Parkinson's disease; APOE, Apolipoprotein E; Aβ42, Amyloid-β 42; T-tau, Total tau; P-tau, Phosphorylated tau; α-syn, α-synuclein; MoCA, Montreal Cognitive Assessment; HVLT, Hopkins Verbal Learning Test; JoLO, Benton Judgment of Line Orientation; LNS, Letter Number Sequencing; SDMT, Symbol Digit Modality Test.

Table S12 Prediction of baseline GFAP and GFAP*time in patients with Amyloid- PD and Amyloid+ PD

Measures	GFAP level				GFAP level*time			
	Amyloid-PD		Amyloid+PD		Amyloid-PD		Amyloid+PD	
	**β**	***p***	**β**	***p***	**β**	***p***	**β**	***p***
Aβ42	0.072	0.235	0.020	0.770	-0.004	0.738	-0.014	0.438
T-tau	**0.133**	**0.032**	**0.478**	**<0.001**	0.013	0.229	-0.026	0.074
P-tau	**0.155**	**0.041**	**0.563**	**0.001**	0.013	0.207	-0.034	0.085
α-syn	**0.179**	**0.027**	**0.344**	**0.001**	-0.013	0.526	0.005	0.828
MoCA	0.005	0.770	-0.002	0.929	**-0.010**	**0.024**	-0.022	0.064
HVLT Total Recall	0.045	0.290	-0.018	0.755	-0.005	0.468	**-0.026**	**0.005**
HVLT Delayed Recall	0.064	0.171	0.013	0.850	-0.006	0.402	**-0.036**	**0.001**
HVLT Retention	0.053	0.177	0.019	0.748	-0.007	0.363	**-0.026**	**0.016**
HVLT Recognition Discrimination	0.018	0.652	-0.010	0.868	-0.006	0.424	-0.017	0.139
JoLO	-0.008	0.758	-0.022	0.611	-0.011	0.092	**-0.022**	**0.001**
LNS	-0.029	0.585	-0.060	0.339	-0.020	0.062	-0.023	0.290
Semantic Fluency Test	-0.006	0.889	-0.053	0.413	-0.005	0.489	**-0.035**	**0.036**
SDMT	0.003	0.482	0.040	0.569	**-0.024**	**0.028**	**-0.067**	**0.038**

The regression coefficients (β) and adjusted p-values computed by generalized linear mixed models.

Adjusted for age, gender, educational level, APOE ε4 carrier status, and disease duration.

The bold emphasis in the table means $p < 0.05$.

Abbreviations: GFAP, glial fibrillary acidic protein; PD, Parkinson's disease; Aβ42, Amyloid-β 42; T-tau, Total tau; P-tau, Phosphorylated tau; α-syn, α-synuclein; MoCA, Montreal Cognitive Assessment; HVLT, Hopkins Verbal Learning Test; JoLO, Benton Judgment of Line Orientation; LNS, Letter Number Sequencing; SDMT, Symbol Digit Modality Test.
